# Pharmacologic targeting of Cdc42 GTPase by a small molecule Cdc42 activity-specific inhibitor prevents platelet activation and thrombosis

**DOI:** 10.1038/s41598-021-92654-6

**Published:** 2021-06-23

**Authors:** Xin Duan, Rehana Perveen, Akhila Dandamudi, Reheman Adili, James Johnson, Kevin Funk, Mark Berryman, Ashley Kuenzi Davis, Michael Holinstat, Yi Zheng, Huzoor Akbar

**Affiliations:** 1 Department of Biomedical Sciences, Heritage College of Osteopathic Medicine, Ohio University, Athens, OH 45701 USA; 2 Division of Experimental Hematology and Cancer Biology, Children’s Hospital Medical Center, University of Cincinnati, Cincinnati, OH 45229 USA; 3 Department of Pharmacology, University of Michigan Medical School, Ann Arbor, MI 48109 USA

**Keywords:** Drug discovery, Molecular medicine

## Abstract

Gene targeting of Cdc42 GTPase has been shown to inhibit platelet activation. In this study, we investigated a hypothesis that inhibition of Cdc42 activity by CASIN, a small molecule Cdc42 Activity-Specific INhibitor, may down regulate platelet activation and thrombus formation. We investigated the effects of CASIN on platelet activation in vitro and thrombosis in vivo. In human platelets, CASIN, but not its inactive analog Pirl7, blocked collagen induced activation of Cdc42 and inhibited phosphorylation of its downstream effector, PAK1/2. Moreover, addition of CASIN to washed human platelets inhibited platelet spreading on immobilized fibrinogen. Treatment of human platelets with CASIN inhibited collagen or thrombin induced: (a) ATP secretion and platelet aggregation; and (b) phosphorylation of Akt, ERK and p38-MAPK. Pre-incubation of platelets with Pirl7, an inactive analog of CASIN, failed to inhibit collagen induced aggregation. Washing of human platelets after incubation with CASIN eliminated its inhibitory effect on collagen induced aggregation. Intraperitoneal administration of CASIN to wild type mice inhibited ex vivo aggregation induced by collagen but did not affect the murine tail bleeding times. CASIN administration, prior to laser-induced injury in murine cremaster muscle arterioles, resulted in formation of smaller and unstable thrombi compared to control mice without CASIN treatment. These data suggest that pharmacologic targeting of Cdc42 by specific and reversible inhibitors may lead to the discovery of novel antithrombotic agents.

## Introduction

Under physiological conditions platelets flow freely in circulation. However, upon vascular injury platelets come in contact with the sub-endothelial extracellular matrix and undergo rapid activation leading to adhesion, shape change, secretion, and aggregation resulting in primary hemostasis. Although this physiologic platelet function is essential for preventing blood loss following a vascular injury, increased platelet reactivity in patients with diseases such as hypertension, hyperlipoproteinemia or diabetes contributes to thrombosis and its potentially fatal complications such as myocardial infarction and cerebral stroke. A number of antithrombotic agents are available for prevention and or management of platelet associated thrombotic complications. However, clinical complications of currently available antiplatelet agents warrant development of safer and more effective antithrombotic therapeutics.

A better understanding of molecular signaling mechanisms involved in platelet activation leading to aggregation and thrombus formation is essential for developing novel antithrombotic agents. Collagen upon interaction with its specific receptors on the platelet surface induces platelet activation^1–4^. Although the roles of heterotrimeric G proteins in platelet activation regulation are well known, low molecular weight monomeric G proteins belonging to Rho family GTPases, namely Rac1^5–8^, Cdc42^9,10^, and RhoA^11^, have emerged as critical regulators of platelet activation^6,12^. Moreover, double Rac1 and Cdc42 knockout mice have been reported to exhibit defective tubulin organization and proplatelet formation^13^. Rac1 has been shown to be involved not only in actin polymerization and stability of platelet aggregates under shear stress^8,14^ but also in secretion and aggregation induced by diverse agonists^7^. Thrombin-induced Rac1-p21-activated kinase (PAK) signaling has been linked to lamellipodia formation^15^ as well as thrombin induced platelet aggregation^5^.

We have reported that platelets from mice with conditional deletion of Cdc42 exhibit diminished activation of PAK1/2, filopodia formation, spreading on immobilized fibrinogen, and reduced secretion and aggregation in response to collagen related peptide^10^. These findings have established that Cdc42-PAK signaling plays a critical role in platelet activation. In this current study, we investigated our hypothesis that if Cdc42 is a key player in platelet activation then its inhibition may down regulate platelet aggregation and thrombus formation.

Cdc42 activity in cells are controlled by guanine-nucleotide exchange factors (GEFs) that catalyze GDP/GTP exchange on Cdc42 to maintain the active Cdc42-GTP pool. We have shown previously that CASIN suppresses Cdc42 activity in a concentration-dependent manner by suppressing GEF catalysis^16,17^. Our findings that CASIN binds only to purified Cdc42 protein but not to Rac1or RhoA have demonstrated that CASIN is a Cdc42-specific inhibitor^18^. Here we report that CASIN inhibits activation of Cdc42 and its effector PAK1/2, as well as platelet actin reorganization, secretion, aggregation and in vivo thrombus formation. Moreover, reversible inhibition of platelet aggregation by CASIN supports the possibility that Cdc42 may serve as a target for developing novel antithrombotic therapies.

## Methods

### Materials

Chemicals and reagents were purchased either from Sigma-Aldrich (St. Louis, MO) or from specifically noted sources. CASIN and Pirl7 were obtained from Chembridge Corporation (San Diego, CA), and purified to greater than 99% by high performance liquid chromatography. Collagen was obtained from Chrono-Log Corporation (Havertown, PA).

Anti-Cdc42 antibody (#2466), PAK1/2/3 (#2604), Phospho-PAK1/2 (#2601, PAK1 Thr423/PAK2 Thr402, p44/42 MAPK (#9102 ERK1/2), Phospho-p44/42 MAPK ERK1/2 (#4370, Thr202/Tyr204), Akt (#9272), Phospho-Akt (#4060, Ser473), p38 MAPK (#8690, Phospho-p38 MAPK (#4511, Thr180/Tyr182), GAPDH (#2118), β-tubulin (#2128) were purchased from Cell Signaling Technology, Danvers, MA. Anti-Rac1 antibody (#05-389) was purchased from Millipore, USA. DyLight 488 anti-GPIb antibody was purchased from Emfret (Eibelstadt, Germany), calcein acetoxymethyl ester (Calcein-AM) was purchased from ThermoFisher (Grand Island, NY). Anti-mouse fibrin antibody was a kind gift from Dr. R. Camire at Children's Hospital of Philadelphia. Anti-fibrin antibody was fluorescently labeled as per manufacturer's instruction using Alexa Fluor 647- antibody labeling kit from ThermoFisher (Grand Island, NY).

### Collection of blood and preparation of washed human platelet suspensions

All experiments using human blood from healthy volunteers were performed according to the protocols approved by the Institutional Review Board at Ohio University (Protocol # 08X126), Athens, Ohio or Cincinnati Children’s Hospital Research Foundation (Protocol # 2010-1855), Cincinnati, Ohio. All methods were performed in accordance with the approved relevant guidelines and regulations. Each volunteer was required to sign an informed consent form approved by the appropriate Institutional Review Board. Procedures for drawing human blood, isolation of platelet-rich plasma (PRP) and preparation of washed platelet suspensions are the same as reported earlier^19,20^. The platelet count was adjusted to 3 × 10^8^ per ml for aggregation studies.

### Mouse maintenance, blood collection, and preparation of platelets

All experiments using mice were performed according to the protocols approved by the Institutional Animal Care and Use Committees at the Children's Hospital Research Foundation (IACUC Protocol#8D06052), Cincinnati, Ohio, or at Ohio University (IACUC Protocol#H08-12) Athens, Ohio, or the University Committee on Use and Care of Animals (UCUCA), University of Michigan. C57BL/6 wild type (WT) mice were purchased from Jackson Laboratories (Bar Harbor, ME, USA). All methods were performed in accordance with the approved relevant guidelines and regulations in the above protocols, and the study was carried out in compliance with the ARRIVE guidelines. Protocols for drawing blood and preparation of platelet rich plasma are essentially the same as we described earlier^7^. For each in vitro experiment, blood was drawn from four DMSO and four CASIN treated mice. Different sets of mice were used for aggregation studies, bleeding times and in vivo thrombosis.

### Cdc42 and Rac1 GTPase activity assay

The relative levels of Cdc42-GTP and Rac1-GTP in washed human platelets were quantified from the same platelet lysates by the effector domain of GST-PAK1 during a pull-down assay as reported earlier^21^. The GTP-bound Cdc42 or Rac1 was quantitatively detected by Western blotting using anti-Cdc42 and anti-Rac1 antibodies.

### Phosphorylation of PAK1/2, ERK, P38-MAPK and Akt

Washed human platelets were stimulated with collagen for a specified time. The reactions were terminated by addition of 4 × sample buffer. Western blotting of p-PAK1/2, p-ERK, p-P38-MAPK, p-Akt, and GAPDH was done as reported earlier^10^.

### Assessment of platelet actin structures on immobilized fibrinogen

Platelet spreading on immobilized fibrinogen was performed as described earlier^11,19^. Glass coverslips were coated with fibrinogen (50 μg/ml) overnight at 4 °C. Non-specific binding sits were blocked by incubating coverslips with 1% bovine serum albumin (BSA, 1%) in phosphate-buffered saline (PBS) at 37 °C. Coverslips were rinsed with Tyrode’s-HEPES buffer after removing BSA. Washed human platelets (100,000) in the presence or absence of CASIN were layered over coverslips and incubated at 37 °C for 10 min. The coverslips were rinsed with PBS to remove free platelets. Platelets on coverslips were then fixed with 4% paraformaldehyde for ten minutes, rinsed twice with PBS, and permeabilized with 0.1% Triton X-100 for 60 s. After two rinses with PBS platelets were stained with Alexa 488-phalloidin to visualize F-actin^10^. The immuno-fluorescence images were taken with a Plan Apo 20x/1.4 objective (Nikon confocal microscope, model 552541 T1-HUBC/A). Digital photos were recorded with NIS elements Image. Platelet spreading was quantified using the NIS elements software.

### Assessment of P-Selectin release, ATP secretion and platelet aggregation

The release of P-selectin from the α-granules was quantified by flow cytometry as described earlier^19^. Briefly, the platelets were isolated from PRP by centrifugation, washed twice and finally resuspended in HEPES-buffered Tyrode’s solution without calcium, pH 7.4 containing 0.2% bovine serum albumin. Washed platelets (1–1.5 × 10^6^) were incubated with 2 micro liters of FITC conjugated anti-CD62P (P-selectin) antibody (BioLegend, #304904) solution for 15 min at 37 °C without stirring. Expression of P-selectin on platelet surface was quantified by flow cytometry (BD LSR II, BD Biosciences) and analyzed by the FACSDiva or FlowJo^22^.

Secretion of ATP from the dense granules was assessed by a luminescence method using a luciferin/luciferase kit from Chrono-Log Corporation^19^. The luciferin/luciferase reagent was added to platelets one minute prior to addition of collagen. Platelet aggregation was monitored by a standard optical density method using a Lumi-Aggregometer from Chrono-Log Corporation.

### Laser-induced cremaster arteriole thrombosis in mice

Laser-induced cremaster arteriole thrombosis injury was assessed by intravital microscopy as described earlier^23,24^. Briefly, 12-week old C57BL/6 WT male mice were anesthetized with ketamine (100 mg/kg)/xylazine (10 mg/kg) via intraperitoneal injection. Trachea tube was inserted to facilitate breathing and jugular vein cannula was established under dissecting microscope to administer anesthetic (Nembutal, 0.05 mg kg^−1^) and other reagents during the experiments. The cremaster muscle was exteriorized on a custom-made surgical tray for intravital microscopy and constantly super perfused with preheated bicarbonate-buffered saline throughout the experiments. Platelets were fluorescently labeled by injecting anti-platelet antibody (DyLight 488 anti-GPIb, 1 µg/g) and fibrin was detected by anti-fibrin (Alexa Fluor 647, 0.3 µg/g) antibody via jugular vein catheter prior to intravital microscopy. Multiple independent thrombi were induced in the arterioles (30–50 µm diameter) in each mouse by a laser ablation system (Ablate! photoablation system; Intelligent Imaging Innovations, Denver, CO, USA). Images of thrombus formation were acquired in real-time under 63X water-immersion objective with a Zeiss Axio Examiner Z1 fluorescent microscope equipped with solid laser launch system (LaserStack; Intelligent Imaging Innovations) and high-speed sCMOS camera. Images of thrombi were analyzed on Slidebook (Intelligent Imaging Innovations Inc., Denver, CO, USA).

### Statistical analysis

Data are expressed as means ± SD or SEM as described in figure legends. A *p* value of < 0.05 indicates statistically significant difference between the control and test samples.

## Results

### Inhibition of Cdc42 by CASIN blocked platelet actin reorganization

**Figure 1:**
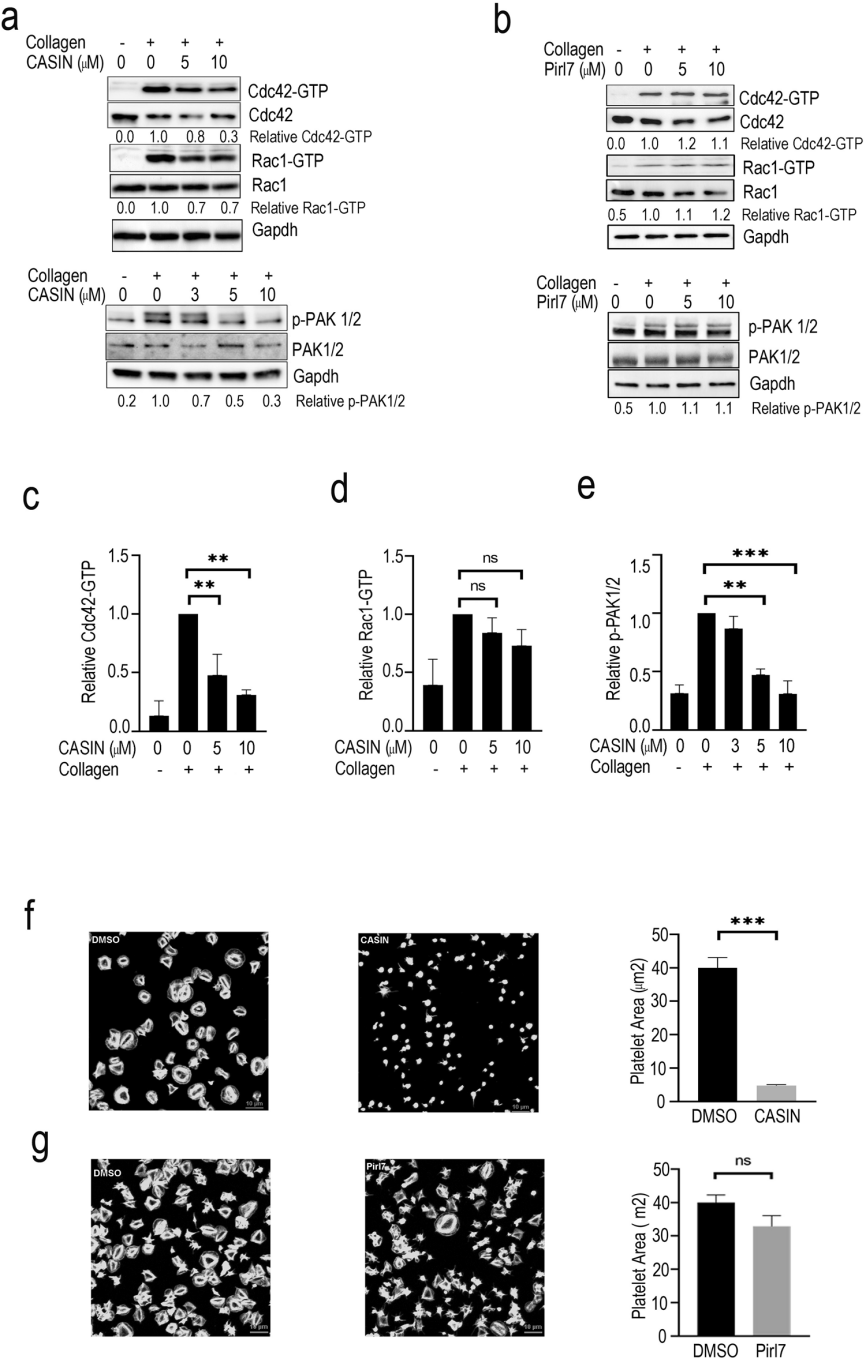
CASIN inhibited activation of Cdc42 GTPase, its effector PAK and spreading of platelets on immobilized fibrinogen. (**a)** Washed human platelets were incubated with collagen (0.3 µg/ml) for two minutes and Cdc42-GTP was quantified. A two-minute pre-incubation of platelets with CASIN inhibited Cdc42-GTP formation and phosphorylation of PAK in a concentration-dependent manner. Total Cdc42 and GAPDH are shown as loading controls. **(b)** A two-minute pre-incubation of washed human platelets with Pirl7, an inactive analog of CASIN, failed to inhibit collagen (0.3 µg/ml) induced Cdc42-GTP activation and phosphorylation of PAK in a concentration dependent manner. Activation of Cdc42 and phosphorylation of PAK were analyzed as described in the methods section. **(c)** Quantifications of Cdc42-GTP and densitometry data. (Mean ± SEM, n = 4, **p < 0.01). **(d)** Quantifications of Rac1-GTP and densitometry data. (Mean ± SEM, n = 4, ns: not significant). **(e)** Bar graphs of PAK1/2 densitometry data. (Mean ± SEM, n = 3, **p < 0.01, ***p < 0.001). **(f and g)** Washed human platelets were prepared from PRP incubated with aspirin (1 mM) for 30 min. CASIN (10 µM), Pirl7 (10 µM) or DMSO was added to platelets containing apyrase (3 U/ml) prior to layering over fibrinogen (50 µg/ml) coated coverslips for 10 min. The coverslips were washed and adherent platelets were processed for immuno-fluorescence microscopy as detailed in the methods section. Platelets treated with CASIN (**f**) as compared to that treated with Pirl7 (**g**) or DMSO (0.1%) (**f,g**) exhibited a significant decrease in spreading on immobilized fibrinogen. Bar graphs quantified platelet spreading on immobilized fibrinogen. (Mean ± SEM, DMSO_f n = 55, CASIN_f n = 57, ***p < 0.001; DMSO_g n = 71, Pirl7_g n = 68, p = 0.065).

To ensure that CASIN acts through Cdc42 signaling, we examined its effect on activation of Cdc42 and its effector molecule p21-activated kinases (PAK) in human platelets. Washed human platelets were incubated with CASIN for two minutes prior to addition of collagen. CASIN, but not its inactive analog Pirl7^18,37^, significantly inhibited collagen induced formation of Cdc42-GTPase in a concentration-dependent manner with a minor effect on Rac1-GTP (Fig. 1a–d; Fig. S1). Activation of Cdc42 has been reported to induce phosphorylation of PAK1/2 in different cell types, including platelets^15,25,26^ and we have shown that agonist induced phosphorylation of PAK1/2 is diminished in platelets from Cdc42 knockout mice^10^. A two-minute treatment of platelets with CASIN, but not Pirl7, inhibited collagen induced phosphorylation of PAK1/2 in a concentration-dependent manner (Fig. 1a,b,e; Fig. S1). These data show that CASIN selectively inhibits activation of Cdc42 and its downstream effector, PAK1/2, in platelets.

Role of Cdc42, in actin polymerization leading to platelet spreading is well established^27–31^. We have shown that platelets deficient in Cdc42 exhibit diminished platelet spreading^10^. To test the effect of CASIN on platelet actin reorganization, we layered aspirin treated washed human platelets over immobilized fibrinogen (50 µg/ml) in the presence of apyrase (3 U/ml) for ten minutes. Platelets treated with CASIN, but not the inactive anolog Pirl7, exhibited significantly less spreading on immobilized fibrinogen as compared to DMSO (Fig. 1f,g). These findings show that inhibition of Cdc42 by CASIN prevents platelet actin reorganization.

### Inhibition of Cdc42 by CASIN suppressed release of P-selectin, secretion of ATP and platelet aggregation

**Figure 2:**
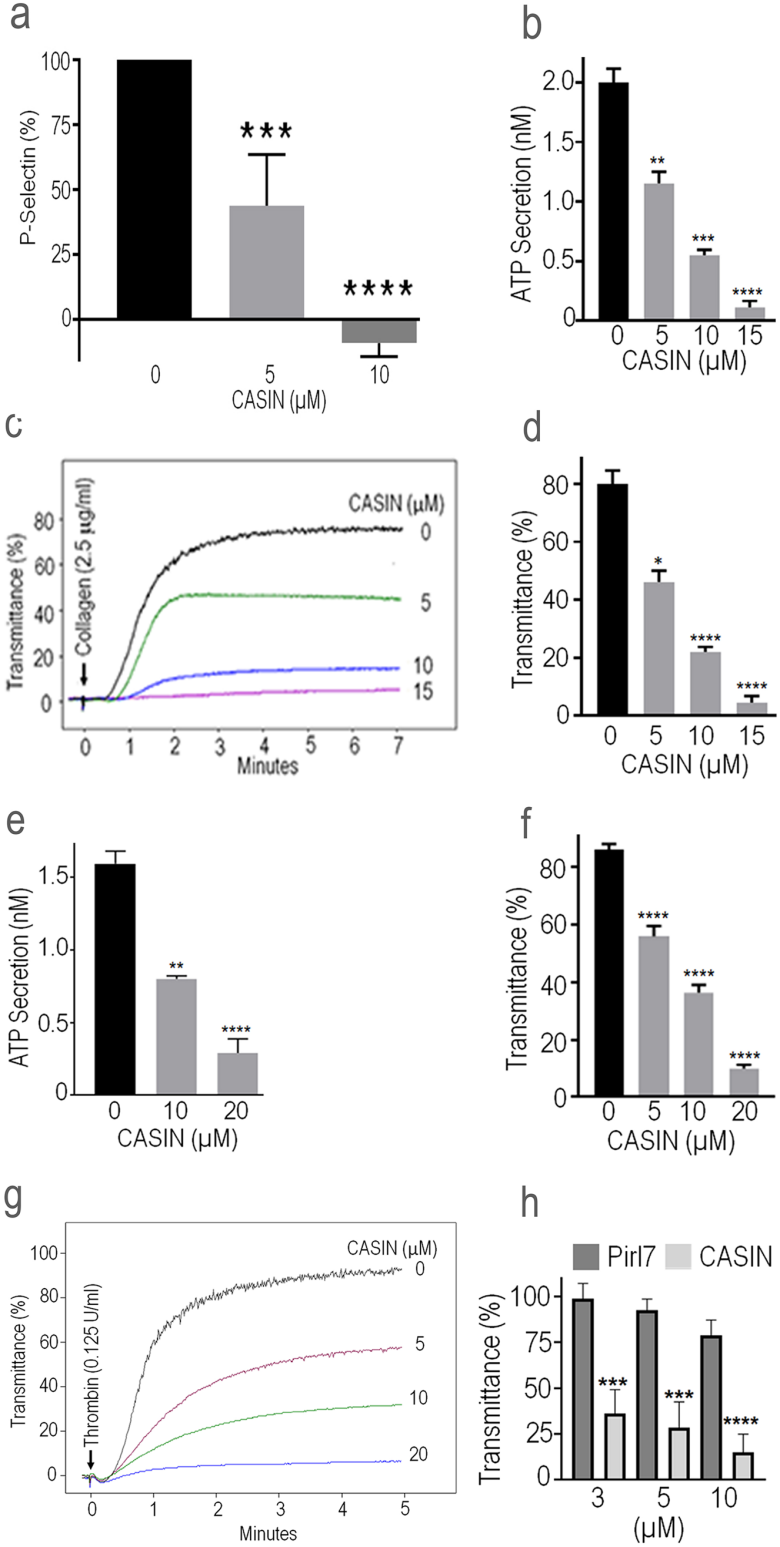
CASIN inhibited P-selectin release, secretion of ATP and platelet aggregation. (**a**) P-selectin was analyzed by flowcytometry as detailed in the Methods. Addition of CASIN (10 μM) to washed human platelets two minutes before stimulation with thrombin (0.1 U/ml) inhibited release of P-selectin (Mean ± SEM, n = 6). (**b, e**) A two minutes pre-incubation of washed human platelets with CASIN inhibited secretion of ATP induced by collagen (Mean ± SEM, DMSO n = 4, CASIN n = 3) or thrombin (Mean ± SEM, DMSO n = 9, CASIN 10 μM n = 4, CASIN 20 μM n = 7) in a concentration-dependent manner**.** Secretion of ATP from platelets was quantified by a luminescence method using luciferin/luciferase kit from the Chrono-Log Corporation. The luciferin/luciferase reagent was added to platelets one minute prior to addition of the agonist. (**c, g**) Addition of CASIN to washed human platelets two minutes prior to stimulation with collagen or thrombin inhibited aggregation in a concentration-dependent manner. (**d, f**) Bar graphs of quantified data of platelet aggregation induced by collagen (Mean ± SEM, DMSO n = 4, CASIN n = 3) or thrombin (Mean ± SEM, DMSO n = 9, CASIN n = 9). **(h)** Platelets were incubated with CASIN or Pirl7 for two minutes before addition of collagen (2.0 μg/ml). Pirl7 did not inhibit collagen induced platelet aggregation. (Mean ± SEM, n = 4). (The p values as shown on bar graphs: *p < 0.05, **p < 0.01, ***p < 0.001, ****p < 0.0001).

Cdc42 has been reported to be involved in regulation of secretion from a variety of cells^32–36^ and we have shown that gene targeting of Cdc42 diminishes release of P-selectin and secretion of ATP from platelets^10^. Here we tested the effect of CASIN on the release of P-selectin and ATP secretion to determine the effect of Cdc42 inhibition on platelet activation. Addition of CASIN to platelets two minutes prior to stimulation with thrombin inhibited the release of P-selectin (Fig. 2a). CASIN added to platelets two minutes before stimulation with collagen or thrombin inhibited secretion of ATP in a concentration-dependent manner (Fig. 2b, 2e). Secretion from platelet granules plays a critical role in propagation of aggregation while inhibition of secretion blocks secondary aggregation^1^. A two-minute pre-treatment of platelets with CASIN inhibited in vitro aggregation induced by collagen or thrombin in a concentration-dependent manner (Fig. 2c,d,f,g). As shown in Fig. 2h, CASIN, but not its inactive analog Pirl7, inhibited collagen induced platelet aggregation.

### CASIN is a reversible inhibitor of platelet activation

**Figure 3:**
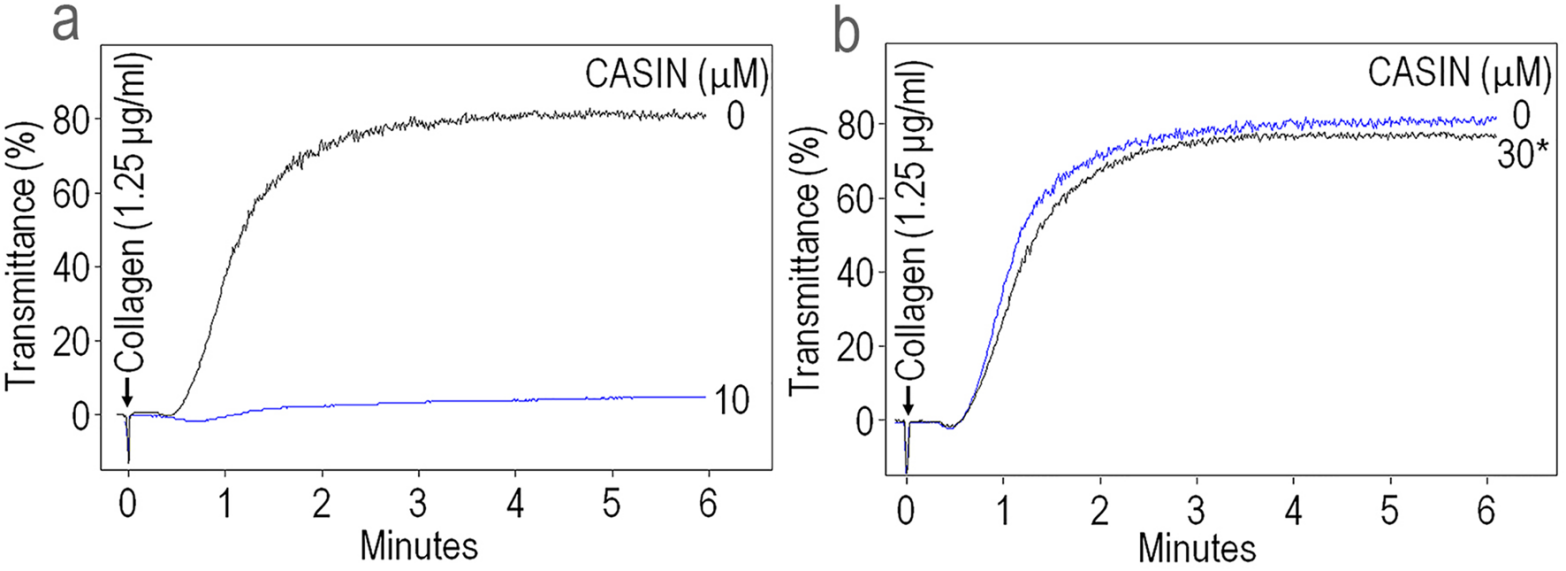
CASIN is a reversible inhibitor of platelet activation. (**a**) Addition of CASIN (10 M$\mu$) to washed human platelets two minutes before stimulation with collagen blocked platelet aggregation. (**b**) Human platelets were incubated with CASIN (30 µM) for 30 min at 37 °C and then washed to remove CASIN prior to addition of collagen. Removal of CASIN restored collagen induced aggregation. The aggregation tracings are representative of four independent experiments.

We investigated whether CASIN is a reversible or an irreversible inhibitor of platelet aggregation. Washed human platelets were incubated with CASIN (30 µM) for 30 min at 37 °C and then washed to remove CASIN. A two-minute pre-incubation with CASIN (10 µM) completely blocked collagen induced platelet aggregation (Fig. 3a) while platelets that were incubated with CASIN (30 µM) and then washed exhibited normal full-scale aggregation in response to collagen (Fig. 3b). These observations suggest that CASIN is a reversible inhibitor of platelet aggregation.

### CASIN blocks phosphorylation of ERK, p38-MAPK and Akt

**Figure 4:**
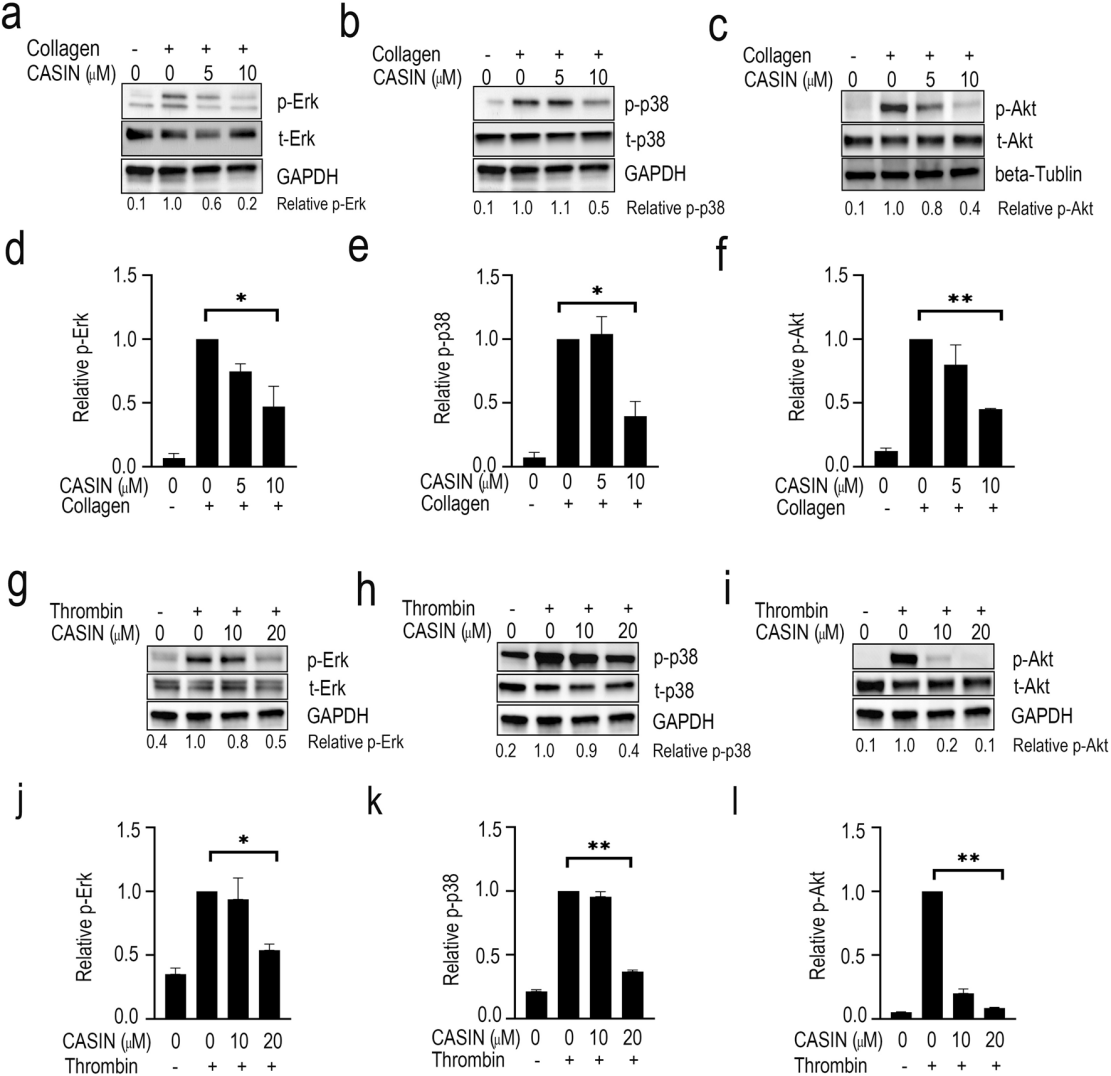
CASIN blocked activation of ERK, p38-MAPK and Akt. CASIN was added to washed human platelets two minutes prior to addition of collagen or thrombin. Samples were incubated at 37 °C with constant stirring in a Chrono-Log aggregometer. The reactions were terminated by adding 4 × sample buffer, processed for Western blotting and probed for the total and phosphorylated ERK (**a** and **g**), p38-MAPK (**b** and **h**), Akt (**c** and **i**) and GAPDH (**a**, **b**, **g**, **h**, **i**) and beta-Tubulin (**c**) as detailed in methods. The GAPDH blot shown in (**a**) is the same as in (**b**); and the GAPDH blot shown in (**g**, **h** and **i**) are the same control. The graphs indicate densitometry analyses using the expression ratios of ERK (**d** and **j**), p38-MAPK (**e** and **k**) and Akt (**f** and** l**) densitometry data. (Mean ± SEM, n = 3, *p < 0.05, **p < 0.01).

The extracellular signal-regulasted kinase (ERK) and p38-MAPK have been linked with agonist induced platelet aggregation^38^. We investigated the effect of CASIN on collagen-induced phosphorylation of ERK and p38-MAPK. A two minutes pre-incubation of platelets with CASIN inhibited collagen (Fig. 4a,b,d,e) or thrombin (Fig. 4g,h,j,k) induced a activation of ERK and p38-MAPK in a concentration-dependent manner. These data show that CASIN inhibits platelet activation, at least in part, by blocking activation of ERK and p38-MAPK.

Platelet stimulation by diverse agonists has been linked to activation of phosphoinositide 3-kinae (PI3K) leading to calcium mobilization, platelet aggregation and thrombus formation^39,40^. PI3K activation leads to phosphorylation of Akt^41–43^. We tested the effect of CASIN on collagen induced Akt phosphorylation in washed human platelets. A two-minute incubation of platelets with CASIN before the addition of collagen (Fig. 4e,f) or thrombin (Fig. 4i,l) blocked phosphorylation of Akt. These findings concur with reported diminished activation of Akt in Cdc42 deficient platelets^10^ and suggest that inhibition of Cdc42 by CASIN, at least in part, blocks platelet aggregation by preventing activation of Akt.

### Administration of CASIN to mice inhibited collagen induced platelet aggregation without affecting the murine tail bleeding time

**Figure 5:**
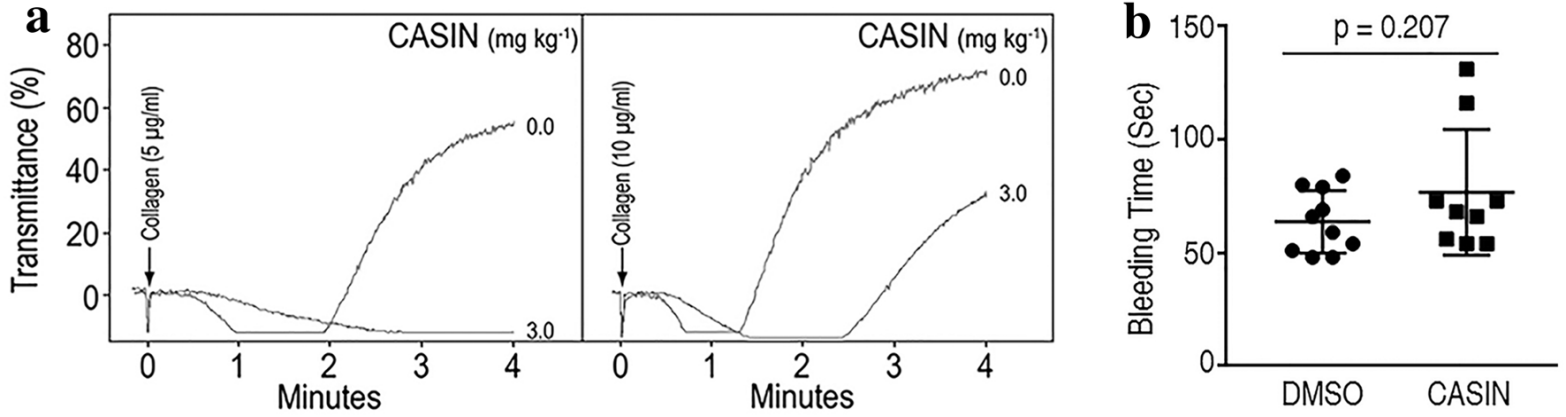
Administration of CASIN to mice inhibited ex vivo platelet aggregation without affecting murine tail bleeding times. (**a**) Intra-peritoneal administration of CASIN (3 mg/kg) inhibited collagen-induced aggregation in citrated platelet rich plasma prepared from four. DMSO and 4 CASIN treated C57BL/6 wild type mice per experiment. Platelet aggregation was monitored by a standard optical density method using a dual channel Lumi-Aggregometer from Chrono-Log Corporation. (**b**) Murine tail bleeding times were assessed 20 min after Intra-peritoneal administration of CASIN (3 mg/kg). CASIN did not affect the tail bleeding times (DMSO n = 10, CASIN n = 9, p = 0.207).

Next, we investigated the effect of intraperitoneal administration of CASIN (3.0 mg/kg) on ex vivo platelet aggregation induced by collagen. This dose was chosen based on our previously reported mouse in vivo data using 2.4 mg/kg for intraperitoneal administration^18^. The half-life of CASIN following intraperitoneal administration was measured to be around two hours^18^. Platelets from mice administered CASIN, when compared to DMSO, exhibited diminished aggregation in response to collagen (Fig. 5a). These findings, taken together with our earlier report that Cdc42 deficiency diminishes platelet aggregation, suggest that CASIN prevents platelet aggregation by blocking the Cdc42 activity.

The possibility that CASIN may prolong hemostatic response by inhibiting platelet aggregation was assessed by monitoring tail-bleeding times as describe earlier^10^ in mice given DMSO or CASIN by intraperitoneal injection. The data in Fig. 5b show that CASIN did not significantly affect the tail bleeding times. This finding shows that CASIN is effective in down regulating platelet activation without adversely affecting the hemostatic response.

### CASIN is effective in suppressing laser induced thrombus formation in vivo

**Figure 6:**
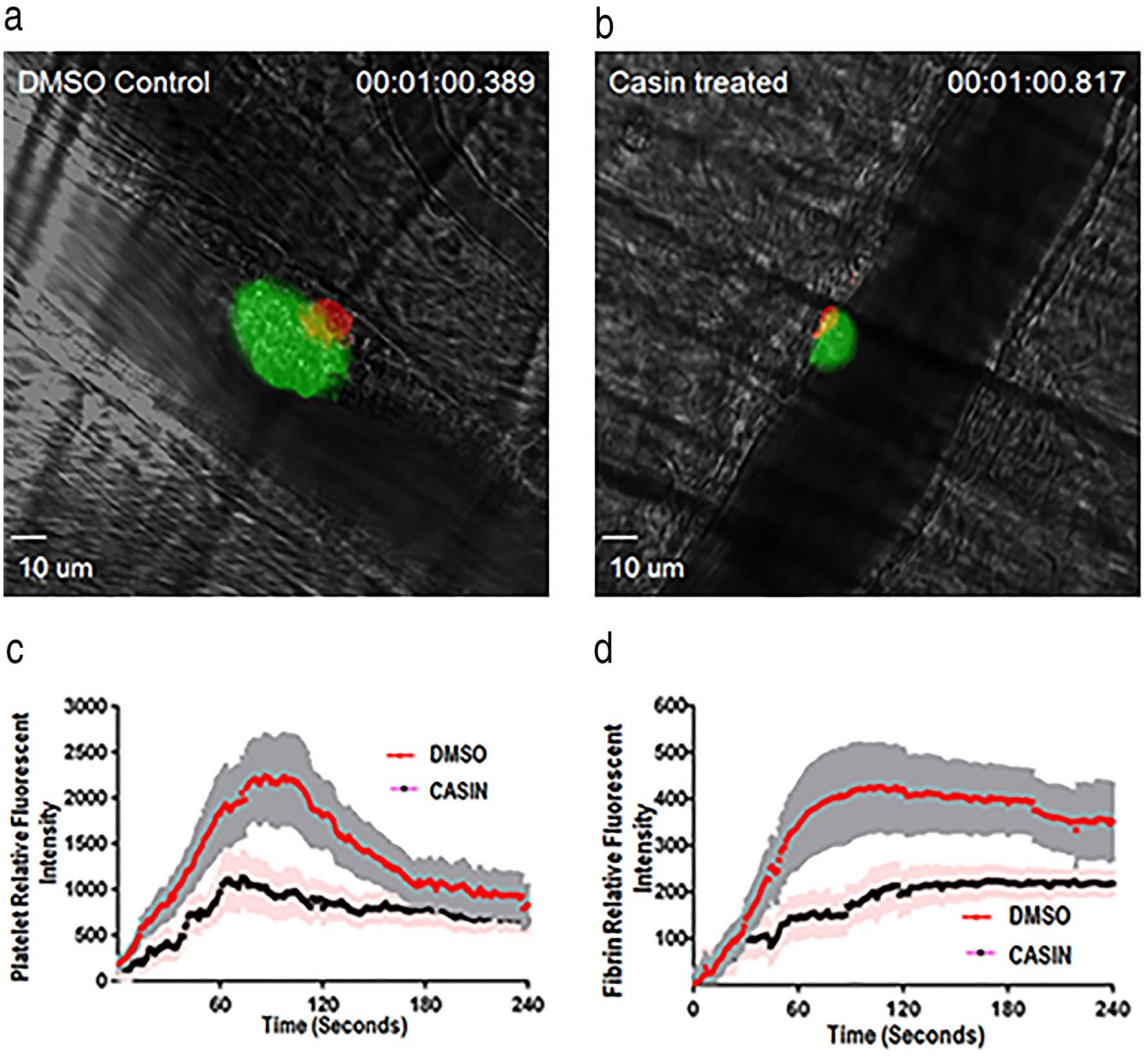
CASIN diminished in vivo thrombus formation. (**a**) Thrombus formation in DMSO (left) or CASIN (right) treated WT mice. CASIN (3.0 mg/kg) or equal volume of DMSO was intravenously injected into mice 10 min prior to induction of thrombus formation by laser-induced injury in the murine cremaster muscle arterioles. Multiple independent thrombi (5- 8 thrombi in each mouse, 3 mice in each group) were induced in the arterioles (30–50 μm diameter) in each mouse by a laser ablation system. Images of thrombus formation were acquired in real-time and analyzed as detailed in methods section. Treated mice, compared to control mice, exhibited a notable decrease in accumulation of platelets (green) and fibrin in the thrombi (red). (**b**) Measurement of fluorescent intensity of platelet accumulation (left) and fibrin deposition (right) at the site of injury in the WT mice in the cremaster arterial thrombosis. The kinetic curves represent the mean fluorescence intensity and the shaded regions are representative of the standard error (SEM). A total of 24 thrombi in DMSO treated mice and 16 thrombi in CASIN treated mice were compared. CASIN treated mice, compared to control mice, exhibited a notable decrease in accumulation of platelets in the thrombi.

To further assess the role of Cdc42 in the in vivo thrombus formation, we investigated the effect of CASIN on thrombus formation in response to laser induced injury in the cremaster muscle arterioles in mice. The dynamic platelet accumulation and fibrin formation in growing thrombi at the site of injury were visualized by intravital microscopy (Fig. 6). Platelets rapidly accumulated on the arteriole wall at the site of laser-induced injury and reached maximal deposition followed by a resolution of the thrombotic clot resulting in a stable thrombus (Fig. 6a,c). In contrast, in CASIN (3.0 mg/kg) treated mice, platelets formed smaller and less stable thrombi at the injury site that were easily washed downstream (Fig. 6b,d). These findings show that inhibiting Cdc42 produces antithrombotic effects and suggest that Cdc42 is a potential target for developing antithrombotic therapies.

## Discussion

We have shown earlier that gene targeting of Cdc42 GTPase diminishes platelet secretion and aggregation^10^. The current study was undertaken to demonstrate that pharmacologic targeting of Cdc42 inhibits platelet aggregation and thrombus formation, therefore Cdc42 GTPase may be a viable target for developing novel antiplatelet agents.

Here we first established that CASIN blocks activation of Cdc42 in platelets (Fig. 1a, c). We found previously that CASIN binds to Cdc42 protein but not other Rho GTPases, e.g. Rac1 or RhoA^18^, and that CASIN inhibits bradykinin induced filopodia formation by Cdc42 but not lysophosphatidic acid (LPA) induced actin stress fiber formation mediated by Rac1 or RhoA in fibroblasts^16,18^. While CASIN seems to affect Rac1-GTP partially in platelet, it is likely an indirect effect due to inhibition of Cdc42 activation^44^. Several other Cdc42 inhibitors have been reported in the literature, including ML141, AZA197, CID-2950007 and secramine^45–48^. Secramine is a non-selective inhibitor^49^ and ZCL278 is an irreversible inhibitor of Cdc42^48^. However, none of these has been subjected to a systemic specificity test (toward other Rho GTPases, Ras, etc.) and in vivo examination as CASIN.

Agonist induced activation of platelets leads to Cdc42 activation^6^. Activated Cdc42 leads to filopodia/lamellipodia formation via p21-activated kinase (PAK)^10,15^ and PAKs are known to connect the Rho GTPase signaling to platelet activation^5^. Our findings that CASIN inhibited not only Cdc42 activity but also phosphorylation of PAK1/2 (Fig. 1a,e). Taken together with the earlier report that platelets from Cdc42 deficient mice exhibit diminished phosphorylation of PAK^10^, these results suggest that CASIN can effectively block activation of Cdc42 and its effector PAK.

The role of Cdc42 in actin polymerization leading to filopodia formation has been demonstrated in numerous cell types including platelets^28,49,50^. Our findings show that platelets layered over immobilized fibrinogen undergo actin polymerization leading to platelet spreading (Fig. 1f,g). Treatment of platelets with CASIN blocked platelet spreading on fibrinogen (Fig. 1f,g). This confirms the role of Cdc42 in platelet spreading and demonstrate that inhibition of Cdc42, as in the case of genetic deficiency of Cdc42^10^, prevents platelet spreading.

Following adhesion to an extracellular matrix and shape change, platelets undergo secretion of granular contents and aggregation. The effect of CASIN on platelet secretion and aggregation was investigated to determine if inhibition of Cdc42 by CASIN would diminish platelet function. Our data indicate that CASIN inhibits the release of P-selectin (Fig. 2a), ATP secretion (Fig. 2b,e) induced by collagen or thrombin in a concentration dependent manner. These findings, taken together with our earlier report showing gene targeting of Cdc42 diminishes platelet activation^10^, suggests that Cdc42 activation plays a critical role in platelet function and CASIN is capable of inhibiting platelet secretion. Secreted secondary mediators such as ADP and thromboxane A_2_ (TXA_2_) play critical roles in the so-called secondary platelet aggregation^1^. CASIN inhibits platelet aggregation (Fig. 2c,d,f,g), at least in part, by blocking the secretion from platelets. Our findings that CASIN, but not its inactive analog Pirl7^18,37^, inhibited platelet aggregation (Fig. 2h) imply that CASIN specifically inhibits Cdc42 GTPase.

Aspirin and most of the ADP receptor antagonists are irreversible inhibitors of platelet activation and as a result, their inhibitory effects last for the life span of platelets. A reversible platelet inhibitor is highly desirable in cases of bleeding episodes or prior to major surgeries. In this study, washing of platelets after incubation with CASIN completely reversed its antithrombotic effect (Fig. 3a,b).

The extracellular kinase (ERK) and p38MAPK have been shown to be involved in platelet activation. The ability of CASIN to block collagen or thrombin induced activation of ERK and p38MAPK (Fig. 4) suggest that inhibition of Cdc42 down regulates platelet activation, at least in part, by inhibiting ERK and P38MAPK. The role of P38MAPK in regulation of thromboxane A_2_ (TXA_2_) synthesis is well known^51^. TXA_2_ is a potent inducer of platelet granular secretion and secondary aggregation^1^ and therefore it is possible that CASIN, at least in part, inhibits secondary platelet activation by inhibiting P38MAPK/TXA_2_ axis of platelet signaling. Platelet stimulation by GPVI mediated signaling induces activation of phosphoinositide 3-kinase (PI3K) isoforms and consequent calcium mobilization, platelet aggregation and thrombus formation^39,40^. Phosphorylation of Akt by PI3K has been linked to irreversible or secondary platelet aggregation^40,52,53^. and platelets genetically deficient in Cdc42 have been shown to exhibit diminished phosphorylation of Akt^10^. Our data that CASIN inhibited collagen or thrombin induced phosphorylation of Akt in platelets (Fig. 4) agrees with the diminished activation of Akt in Cdc42 deficient platelets and suggest that inhibition of aggregation, at least in part, may be due to inhibition of Akt activation. Our findings that CASIN inhibits CRP induced platelet aggregation in aspirin and apyrase treated platelets (see Fig. S3 in supplemental information) further suggest that Cdc42 regulates platelet activation, at least in part, by a TXA_2_-independent mechanism.

The antithrombotic potential of targeting Cdc42 was further investigated by testing the effects of CASIN on ex vivo platelet aggregation and in vivo thrombus formation using the laser induced injury model of thrombosis^23,24,54^. Our observations that CASIN inhibited collagen induced ex vivo platelet aggregation (Fig. 5a) without affecting the tail bleeding times (Fig. 5b) imply that down regulation of Cdc42 may be an effective approach for diminishing platelet aggregation. Two of the CASIN treated mice exhibited longer bleeding times than the DMSO treated mice (Fig. 5b). Given that the murine tail bleeding times are sometimes very variable it is possible that some more mice may exhibit prolonged bleeding times in a larger study. We have shown that deletion of Cdc42 prolongs bleeding times. However, pharmacologic targeting of Cdc42 by CASIN did not prolong the murine tail bleeding times (Fig. 5b). It is possible that the effect of a complete and permanent deletion of Cdc42 is more profound than the effect of a single intraperitoneal dose of a reversible inhibitor of Cdc42. In addition, Cdc42^−/−^ mice exhibit a significantly diminished platelet count^9,10^ whereas pharmacologic inhibition of Cdc42 by CASIN has no effect on platelet count (Fig. S4 in supplemental information). It is possible that the combined effect of the defective platelet function and a diminished platelet count contributes to the prolonged bleeding times in Cdc42^−/−^ mice. Further, our findings that CASIN diminished the size of thrombus by inhibiting platelet aggregation but did not abolish the thrombus formation at the site of injury (Fig. 6) suggest that the hemostatic response required to consolidate the platelet plug to arrest bleeding remains viable.

We have shown earlier that gene deletion of Cdc42 and Rac1 exhibit similar platelet defects namely inhibition of secretion and aggregation^7,10^. Both Cdc42 and Rac1 appear essential for the regulation of ERK and p38MAPK activities mediated by multiple agonist induced signaling. Detailed molecular mechanisms how Cdc42 may interact with or differ from Rac1 signaling in platelets remains to be investigated. Pharmacologic inhibition of Rac1 by NSC23766 prolongs tail bleeding times^7^ but CASIN, a Cdc42 inhibitor, at the tested dosage and conditions did not prolong tail bleeding times (Fig. 5b). This difference may reflect distinct pharmacological properties of NSC23766 and CASIN including pharmacodynamics and pharmacokinetic differences.

In summary, the ability of CASIN to inhibit platelet secretion, aggregation and diminish the size of thrombus induced by laser ablation (Fig. 6) further confirms that Cdc42 plays a critical role in platelet-associated thrombosis and hence may serve as a target for developing novel antithrombotic agents.

## Supplementary Information

Supplementary Information.
