# Supplementary material for: Pharmacologic targeting of Cdc42 GTPase by a small molecule Cdc42 activity-specific inhibitor prevents platelet activation and thrombosis
Source: Sci Rep. 2021 Jun 23;11:13170. doi: 10.1038/s41598-021-92654-6 (PMC8222210; doi:10.1038/s41598-021-92654-6)
Supplement: Supplementary file 1 — Supplementary Information. [file 41598_2021_92654_MOESM1_ESM.docx]

**Supplementary information**

**Pharmacologic targeting of Cdc42 GTPase by a small molecule Cdc42 activity-specific**

**inhibitor prevents platelet activation and thrombosis**

Xin Duan^2+^, Rehana Perveen^1+^, Akhila Dandamudi^2^, Adili Reheman^3^, James Johnson^2^, Kevin Funk^1^, Mark Berryman^1^, Ashley Kuenzi Davis^2^, Michael Holinstat^3^, Yi Zheng^2*^, and Huzoor Akbar^1*^

^1^Department of Biomedical Sciences, Heritage College of Osteopathic Medicine, Ohio University, Athens, OH 45701

^2^Division of Experimental Hematology and Cancer Biology, Children’s Hospital Medical Center, University of Cincinnati, Cincinnati, OH 45229

^3^Department of Pharmacology, University of Michigan Medical school, Ann Arbor, MI 48109

Running title: Cdc42 GTPase as an antithrombotic target

* Correspondence: Huzoor Akbar, Department of Biomedical Sciences, Heritage College of Osteopathic Medicine, Ohio University, Athens, OH 45701; Tel: 740-591-2801; Email: [Akbar@ohio.edu](mailto:Akbar@ohio.edu); Yi Zheng, Division of Experimental Hematology and Cancer Biology, Children’s Hospital Medical Center, Cincinnati, OH 45229; Tel: 513-636-0595; E-mail: yi.zheng@cchmc.org

^+^ Equal contributions

**
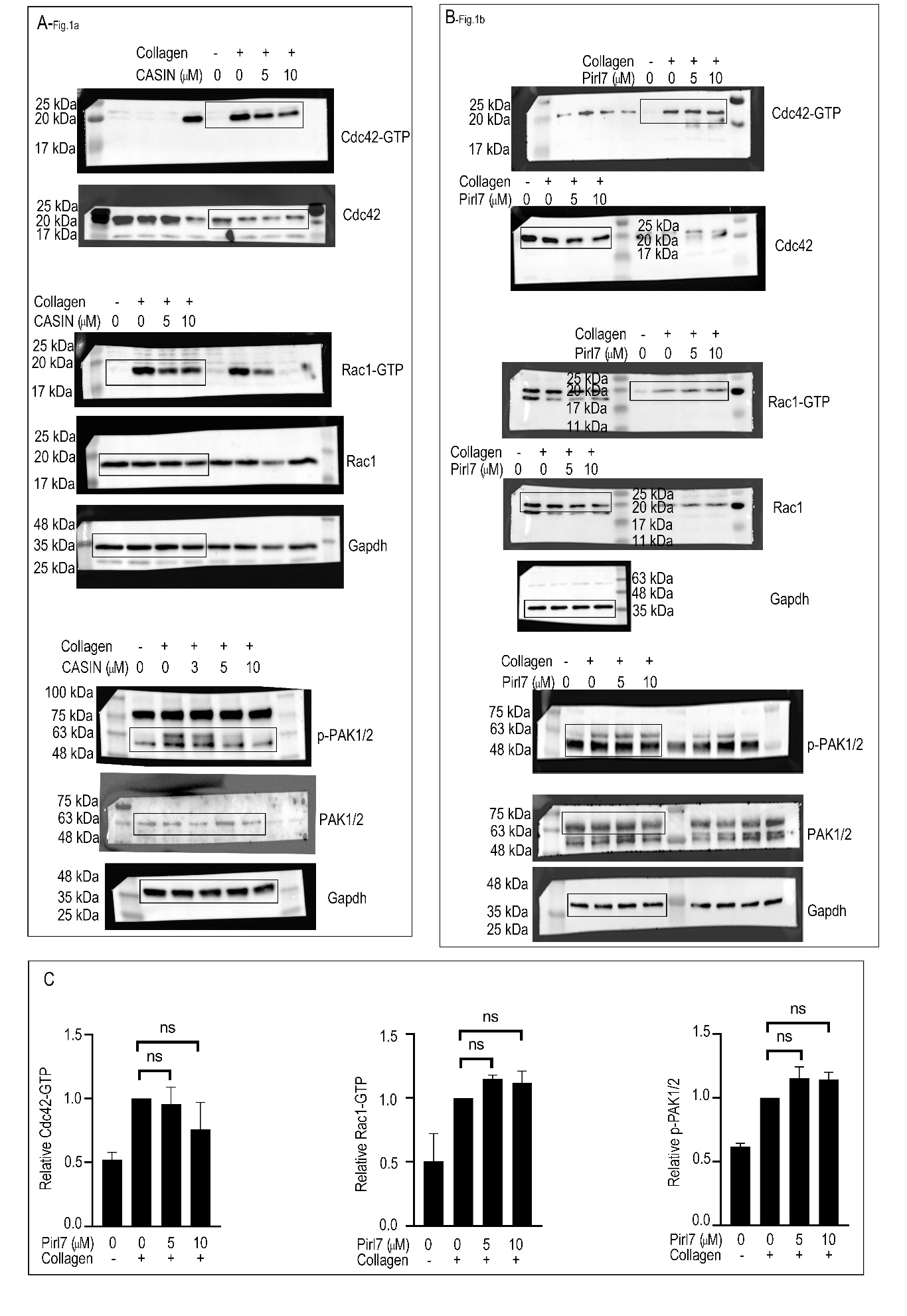
**

**Figure S1. Full Western blots for Figure 1 and quantification of Pirl7 effects.** A) Western blots from Figure 1a showing Cdc42 and Rac1 GTPase activities and p-PAK1/2 and PAK1/2 levels. Boxes show selected bands for the main Figure 1a. B) Western blots from Figure 1b showing Cdc42 and Rac1 GTPase activities and p-PAK1/2 and PAK1/2 levels with or without Pirl7 treatment. Boxes show selected bands for the main Figure 1b. C) Densitometry quantifications of relative Cdc42-GTP, Rac1-GTP, and p-PAK1/2 of the Western blots shown in Figure 1b, with or without Pirl7 treatment (Mean + SEM, n=4, p>0.05).

**
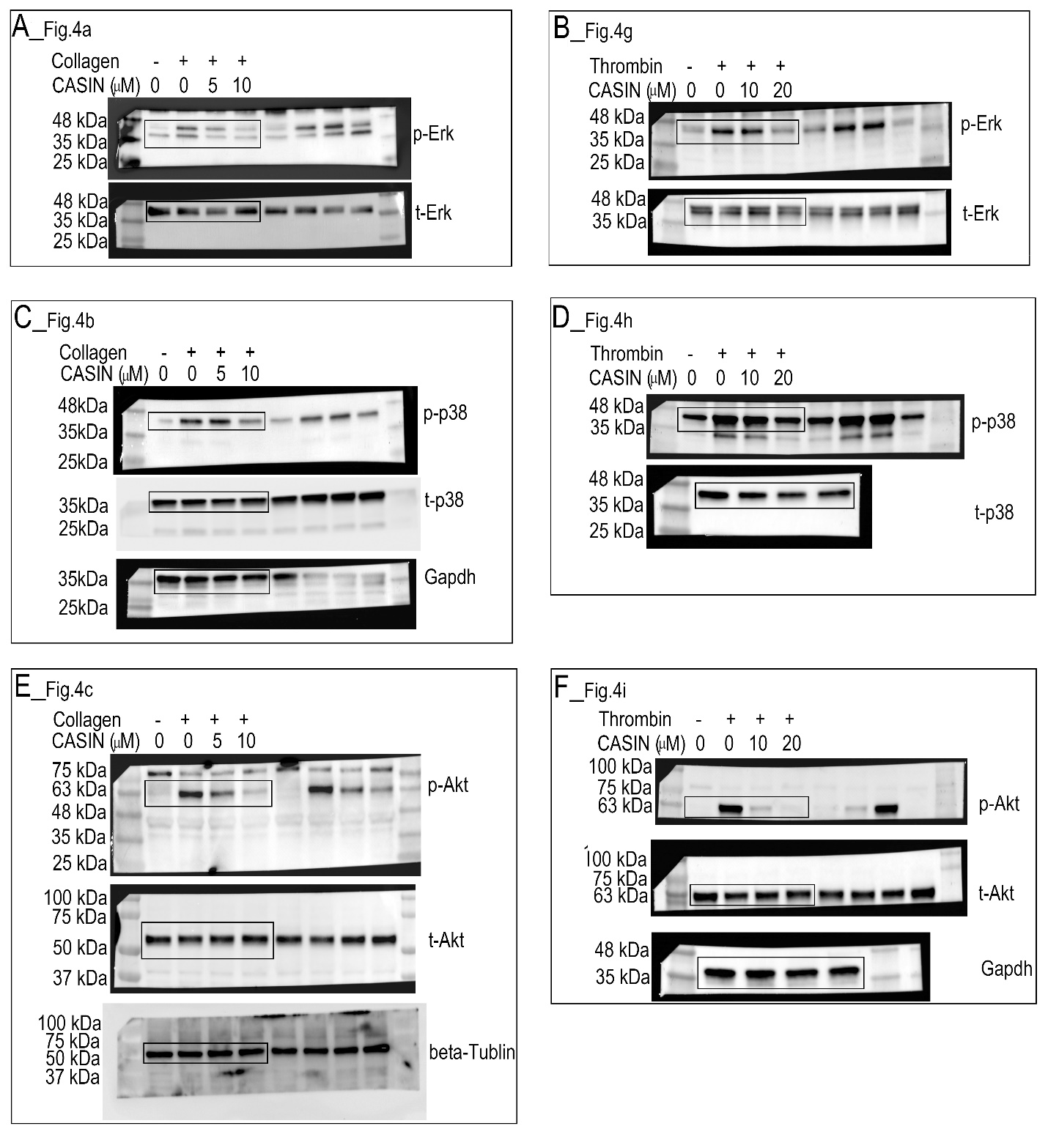
**

**Figure S2. Full Western blots for Figure 4.** A) Western blots of p-Erk and t-Erk shown in Figure 4a. B) Western blots of p-Erk and t-Erk in Figure 4g. C) Western blots of p-p38, t-p38 and Gapdh in Figure 4b. D) Western blots of p-p38 and t-p38 in Figure 4h. E) Western blots of p-Akt, t-Akt and β-Tublin for Figure 4c. F) Western blots of p-Akt, t-Akt and GAPDH for Figure 4i. The GAPDH blot shown in Figure 4g, h and i are the same control. Boxes show selected bands for the main Figure 4a-c and Figure 4g-i.

**
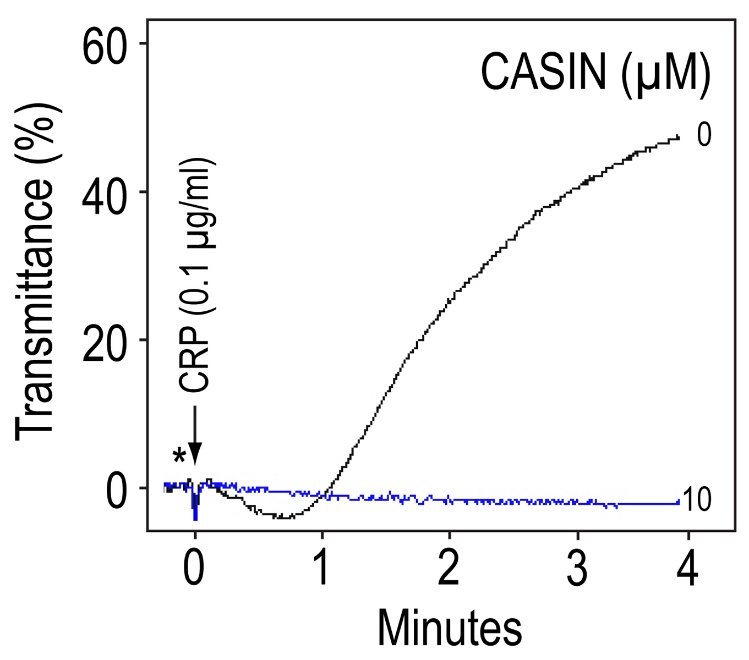
**

**Figure S3. CASIN inhibited CRP induced aggregation in aspirin and apyrase treated platelets.** Washed human platelets were incubated with Aspirin (1 mM) and apyrase (3 U/ml) prior to addition of DMSO (0.1%) or CASIN (10 μM). CRP induced platelet aggregation was monitored by a standard optical density method using a Lumi-Aggregometer from Chrono-Log Corporation, Havertown, PA, USA. The aggregation tracings are a representative of four independent experiments.

**
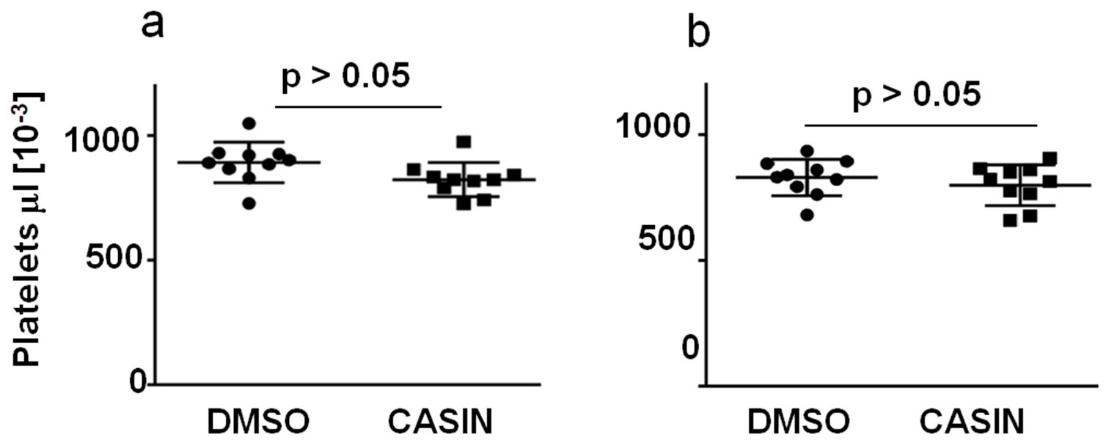
**

**Figure S4. CASIN administration does not affect the murine blood platelet count.** Blood was drawn from C57/BL6 wild type mice after (a) 20 minutes and (b) 2 hours of intraperitoneal injection of DMSO (n=10) or CASIN (3.0 mg/kg, n=10). CASN administration, as compared to DMSO, did not alter the platelet count after 20 minutes (p>0.05) or 2 hours (p>0.05). Platelets were counted using a Hemavet 950FS (Drew Scientific, CT, USA).
